# Characteristics of obstetric urogenital fistulas in a regional teaching hospital in Burkina Faso: a retrospective cross-sectional study

**DOI:** 10.11604/pamj.2023.44.105.35733

**Published:** 2023-02-24

**Authors:** Tiéoulé Mamadou Traore, Souleymane Ouedraogo, Moussa Kabore, Salam Ouedraogo, Joël Judicaël Traore

**Affiliations:** 1 Department of Urology and Andrology, Regional Teaching Hospital of Ouahigouya, Ouahigouya, Burkina Faso,; 2 Department of General Surgery, Regional Teaching Hospital of Ouahigouya, Ouahigouya, Burkina Faso

**Keywords:** Obstetric fistulas, prolonged labor, Burkina Faso

## Abstract

**Introduction:**

Obstetric fistula (OF) remains a major public health problem in low-income countries. This study aimed to investigate the sociodemographic, clinical, and therapeutic characteristics of obstetric urogenital fistulas in a regional teaching hospital in Burkina Faso.

**Methods:**

a retrospective cross-sectional study from 1^st^ January 2015 to 31^st^ December 2019 included 50 women who underwent OF surgery repair in the regional teaching hospital of Ouahigouya in Burkina Faso. Case identification was completed by self-reported constant urine leakage and was confirmed by clinical assessment. Data on socio-demographic, clinical, and therapeutic characteristics have been collected from the hospital medical records and analyzed.

**Results:**

the mean age of the patients was 29.40 ± 9.4 years (range 15 -55 years). The majority of patients were in the age group between (15-25) years old (44%). Forty-three patients (86%) were residing in rural areas and forty-seven patients (94%) were housekeepers. Twenty-six patients (52%) were primiparous. The majority of patients had received no prenatal care 29 (58%). The majority of patients had a spontaneous vaginal delivery 36 (72%). The duration of labor was greater than 48 hours in 31 (62%) patients. Vesicovaginal fistulas (VVF) accounted for 80% of cases. Ten (20%) patients had previously undergone surgery for the same fistula. The mean size of the fistulas was 1.8±1.4 cm (range 0.5 - 6 cm). At three months of follow-up, the successful closure rate was 68%. Sixteen (32%) patients have experienced a failure of fistula closure.

**Conclusion:**

the majority of fistula survivors were women of reproductive age who were living in rural areas and housekeepers. Mothers having no antenatal care, and having prolonged labor were at increased risk of developing OF. The majority of fistulas were simple fistulas and the most common type of OF was VVF. Surgical outcomes showed a high failure rate.

## Introduction

Obstetric fistula (OF) is an abnormal connection between the urinary tract and/or anorectal tract and the genital tract due to prolonged obstructed labor, leading to a continuous loss of urine and/or stool through the vagina [1]. OF is a major public health problem in low-income countries [2]. Each year, between 50,000 to 100,000 women throughout the world are affected by OF and about 2 million women currently live with untreated fistula [3]. In sub-Saharan Africa, OF prevalence is estimated at 1.6 per 1000 women of reproductive age [4]. In Burkina Faso, the incidence of OF was 23.1 per 100,000 births [5]. The psychological consequences of OF are devastating. Women suffering from OF are victims of stigma and social isolation. Previous studies have shown that OF mostly affects young rural women of reproductive age after prolonged obstructed labor [6-8]. Vesicovaginal fistula (VVF) is the most common type of urogenital fistulas [7]. The management of OF is mainly surgical. Generally, OF repair has a higher surgical closure rate when performed by skilled surgeons [7,8].

The characteristics of OF in the regional teaching hospital of Ouahigouya in Burkina Faso are not known, despite the importance of the issue. What are the socio-demographic, clinical, and therapeutic characteristics of OF patients treated in our regional teaching hospital? This study aimed to investigate the socio-demographic, clinical, and therapeutic characteristics of OF patients treated in our regional teaching hospital in Burkina Faso.

## Methods

**Study design and period:** a retrospective cross-sectional study over five years, from 1^st^ January 2015 to 31^st^ December 2019 has been carried out in the regional teaching hospital of Ouahigouya in Burkina Faso. Case identification was completed by self-reported constant urine leakage and was confirmed by clinical assessment. Women who underwent OF surgery during the study period were included in the study. Patients with fistulas caused by other mechanisms (congenital fistula, traumatic fistula, cancer-related fistula, iatrogenic fistula) were not included in the study. Patients with less than three months of follow-up and those with missing data were excluded from this study.

**Definition of variables:** Obstetric fistula (OF) is due to prolonged obstructed labor with a fistula from the urinary tract and/or anorectal tract to the genital tract caused by ischemia and necrosis [1]. The patients' socio-demographic and obstetric characteristics studied were: patients' age at marriage, age at the time of fistula occurrence, residence, weight, height, parity, marital status, prenatal care, duration of labor, and mode of delivery (cesarian section, vaginal delivery). The fistula characteristics studied were: location, size of the defect (cm), presence of fibrosis, previous repair, type of surgery, duration of leakage, and type of fistula according to Dakar's classification [8].

Dakar's classification is based on the involvement of the continence system (type I: continence system is not involved, type II: continence system is involved without transsection, type III: continence system is involved with transsection). A dye test was performed to assess the surgical outcome at postoperative three months. Two surgeons have performed all the fistula surgeries: a general surgeon and a urologist surgeon.

**Analysis and measures:** all statistical analyses were performed using Statistical Package for the Social Sciences (SPSS) software in version 21.0. Qualitative variables were presented in terms of numbers and percentages. Quantitative variables were presented as mean, with their standard deviation (SD), maximum (Max), and minimum (Min). Patients were assessed at three months. Three surgical outcomes were considered: unclosed fistula, closed fistula without residual urinary incontinence and closed fistula with residual urinary incontinence.

**Ethics:** the Ethics Research Committee of the regional teaching hospital of Ouahigouya in Burkina Faso approved this project, which was carried out in accordance with the Declaration of Helsinki.

## Results

During the study period, 50 patients who underwent OF surgery had been included. No patients were excluded from this study. The annual frequency of OF was 10 cases. The mean age of the patients at the time of fistula occurrence was 29.40 ± 9.4 years (range 15-55 years). The majority of patients were in the age group between (15-25) years old (44%). Forty-three patients (86%) were residing in rural areas and forty-seven patients (94%) were housekeepers. Twenty-six patients (52%) were primiparous. The socio-demographic data of women are presented in Table 1.

**Table 1:** socio-demographic characteristics of women (N=50)

Variables	Mean ± SD or N (%)
**Age** (years)	29.4±9.4 (range 15-55)
**Age at the time of marriage** (years)	17.9±6.5 (range 16- 24)
**Residence**	
Rural	43 (86)
Urban	7 (14)
**Profession**	
Housekeeper	47 (94)
Student	2 (4)
Civil servant	1 (2)
**Marital status before fistula**	
Married	46 (92)
Unmarried	4 (8)
**Parity**	
Primipare	26 (52)
Multipare	10 (20)
Paucipare	14 (28)
Weight (kg)	56.4±7.4 (range 44-75)
Height (m)	162±1.3 (range 148-179)

**Abbreviation:** SD, Standard Deviation.

**Figure 1:**
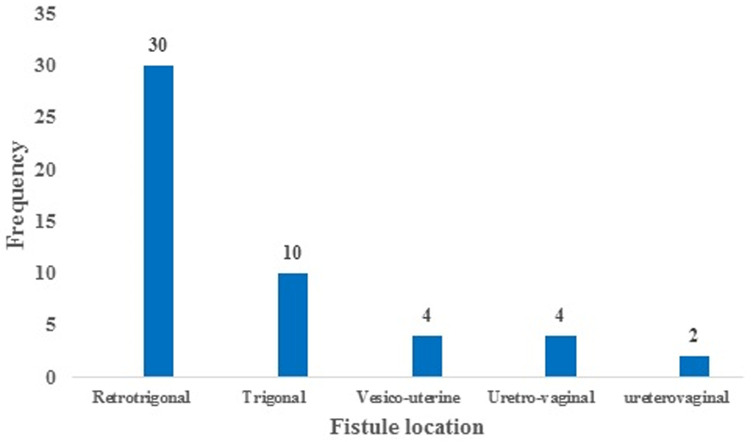
distribution of patients according to fistula location

Of the 50 patients, 29 (58%) had received no prenatal care, 43 (86%) had given birth in a health care center and 7 (14%) had given birth at home without any medical assistance. The duration of labor was greater than 48 hours in 31 (62%) patients. Thirty-six (72%) women had a spontaneous vaginal delivery. The majority of fistulas were VVFs 40 (80%). VVF was retro-trigonal in 30 cases and trigonal in 10 cases (Figure 1). Three patients (6%) had an associated rectovaginal fistula. The mean size of the fistulas was 1.8±1.4cm (range 0.5- 6cm). The mode of delivery and the clinical characteristics of fistulas are presented in Table 2.

**Table 2:** mode of delivery and fistula characteristics (N=50)

Variables	Mean ± SD or N (%)
**Duration of labor** (hours)	
<24	6 (12)
24-48	13 (26)
>48	31 (62)
**Mode of delivery**	
Spontaneous vaginal birth	36 (72)
Instrumental vaginal birth	8 (16)
**Emergency cesarean birth after prolonged labor**	6 (12)
Size of defect (cm)	1.8±1.4 (range 0.5-6)
**Type of Dakar’s classification**	
I	42 (84)
II	4 (8)
III	4 (8)
**Duration of leakage (years)**	
<1	35 (70)
1-5	10 (20)
5-10	5 (10)
**Fibrosis**	
Yes	20 (40)
No	30 (60)
**Previous repair**	
Yes	10 (20)
No	40 (80)

**Abbreviation:** SD, Standard Deviation.

The transvaginal repair had been used in 42 (84%) patients, transabdominal repair in 5 (10%) patients and a combined approach in 3 (6%) patients. Chassar-Moir technique had been used to repair all the VVFs. No Martius flap was used. Three patients (6%) had undergone associated procedures such as recto-vaginal fistula closure. The average length of hospital stay was 13.30 ± 4.70 days (range 3-21 days). The three-month outcome of fistula surgery was: closed fistula without residual urinary incontinence in 28 (56%) patients, closed fistula with residual urinary incontinence in 6 (12%) patients, and unclosed fistulas in 16 (32%) patients. Fistula closure was achieved in 34 (68%) patients.

## Discussion

The present study aimed to investigate the socio-demographic, clinical, and therapeutic characteristics of OF patients treated in the regional teaching hospital of Ouahigouya in Burkina Faso. The annual frequency of obstetric fistula was 10 cases. Our study showed that OF mainly affects women of reproductive age, residing in rural areas and most often primiparous with a lack of antenatal care. Additionally, OF more occurs after a spontaneous vaginal delivery with prolonged obstructed labor. The most common fistula type was VVF, with a high failure rate after surgery.

The annual frequency of 10 cases found in this study is lower than that reported by others authors in the same country. Kaboré *et al*. reported an annual frequency of 69 cases and Kambou *et al*. 28.5 cases [9,10]. The study by Kaboré *et al*. was multicenter, including seven fistula centers in Burkina Faso [9]. The incidence of OF is underestimated because many women do not seek care and think that fistula is a normal curse. More recently, a growing incidence of iatrogenic OFs has been reported in developing countries. However, it is unclear whether these fistulas are of iatrogenic obstetric origin or caused by ischemia resulting from obstructed labor [11]. In the present study, six fistulas occurred after emergency caesarian section for prolonged obstructed labor. We considered these fistulas as OFs because there were no iatrogenic lesions identified intraoperatively during the caesarian section. So these fistulas are caused by ischemia resulting from obstructed labor.

The socio-demographic profile of women suffering from OF in the present study is consistent with data reported in the literature [12,13]. OF affects mostly young women of reproductive age. In the present study, the patients' mean age at the time of fistula occurrence was 29.4 years. The majority of patients were in the age group between (15-25) years old (44%). This finding is similar to that reported by Nsambi *et al*. in Congo [12]. In a case-control study designed to identify risk factors for OF, Tilahun *et al*. showed that age at the pregnancy of < 18 years, residing in rural areas, lack of antenatal care, no history of modern contraception utilization, post-term pregnancy and duration of labor > 24 hours were associated with OF [12,13]. In the present study, 86% of the patients live in rural areas. This finding is consistent with that of Tilahun *et al*. who showed that 81.2% of women with OF live in rural areas [13]. In these rural areas, women's access to quality obstetrical care is limited. The remoteness of health centers and the refusal of some men to allow their wives to attend health centers are barriers to providing quality obstetric care. In the present study, 58% of the patients had received no prenatal care despite free healthcare for pregnant women instituted by the government.

Home birth remains a very common practice in our context. In the present study, 14% of women had given birth at home without medical assistance. The home birth rate reported by Nsambi *et al*. was 5 times higher than that reported in the present study [12].

The duration of labor is a strong predictor of OF. In the present study, 62% of patients had a labor duration of more than 48 hours. Many women attempt home birth, and end up going to a health care center because of dystocia and the inability to deliver. The unrelenting pressure of the entrapped fetal head against the mother's pelvis during prolonged labor can cut off the flow of blood to the soft tissues of the bladder, vagina, and rectum. Regardless of fetal outcome, prolonged obstructed labor usually results in sloughing away of injured pelvic tissue, leaving a fistula between adjacent organs [14]. In a clinical review of the risk factors for OF, Tebeu *et al*. estimated that 20% to 95.7% of patients with OF had a labor duration of more than 24 hours [6].

Prolonged labor is a preventable risk factor that should not be seen nowadays. OF reflects the quality and the level of perinatal care delivered by the healthcare system [15]. In the present study, 52% of the women were primiparous. This finding is consistent with that reported by Nsambi *et al*. (90.9%) and Hawkins *et al*. (54.5%) [12,16]. Majority of OF occur in primiparous women due to cephalopelvic disproportion and prolonged labor [17,18].

The most common type of OF in the present study was VVF (80%). This finding is consistent with the data in the literature [12,19]. Nsambi *et al*. and Egziabher *et al*. reported respectively rates of 96.3% and 70.6% for VVF [12,19]. The close anatomical relationship between the bladder and the vagina explains this predominance of VVF. Of the 50 patients, 10 (20%) underwent previous surgery for the same fistula. Egziabher *et al*. found that 14% of patients underwent previous surgery for the same fistula [19]. Prior surgery is known to be a predictor for fistula failure repair [20,21]. Indeed, after each attempt, vaginal scarring increases reducing viable tissues.

Chassar-Moir technique has been used to repair all VVFs in the present study. Diallo *et al*. have used Chassar-Moir technique in 63% of cases [22]. It consists of excising the perifistulous fibrous tissue to obtain healthy tissue before closing the bladder and vagina. The disadvantage of excising the fistula tract is the increase of fistula size, the risk of bleeding and and the risk of ureteral injury. This technique is used mostly to repair simple fistulas [22]. In the present study, 84% of fistulas were simple fistulas. Therefore, a high success rate could be expected with the Chassar-Moir technique in the present study. Adherence to basic principles, such as pre-operative evaluation, adequate exposure of the fistula, good hemostasis, resection of devascularized tissue, excision of surrounding fibrous tissue and removal of foreign body, tension-free closure, and adequate postoperative urinary drainage, is the most important factor for successful fistula repair [14]. In addition, the use of Martius flap in some cases could improve our postoperative results.

Analyzing the surgery outcomes in the present study, we found that the fistula closure rate was 68% after three months of follow-up. However, the failure rate (32%) in the present study is high compared to that reported previously. Kabore *et al*. reported a closure failure rate of 11.4% [23]. In a systematic literature review, Hillary *et al*. have estimated the average successful closure rate 87% (range 58-100%) [7]. It should be recognized that the closure failure rate (32%) is very high in the present study. Indeed, our department does not have a solid experience in the management of OF. Cromwell *et al*. estimate that limited surgical experience would seem to make the failure of a repair more likely [24]. Collaboration with the teams of Kambou and Kaboré, who have extensive experience in the management of urogenital fistulas in Burkina Faso, could help improve our results [10]. The rate of residual urinary incontinence in the present study (12%) is similar to that reported in the literature [7,25]. Hillary *et al*. estimated the rate of residual urinary incontinence at about 10% [7]. This residual urinary incontinence impairs the patient's quality of life and remains a challenge for the surgeon.

This study has some limitations. The first limitation was the small sample size. Only 50 patients have been enrolled in this study. Also, this study didn't identify the risk factors for failure to close fistula. Additionally, we have the moncenteric and retrospective nature of this study. The next study will aim to identify the risk factors for failure to close fistula. Despite the limitations, this study addressed our research question.

## Conclusion

The majority of fistula survivors were women of reproductive age who were living in rural areas and housekeepers. Mothers having no antenatal care, and having prolonged labor were at increased risk of developing obstetric fistula. Thus, delaying the age of first pregnancy, and improving access to basic obstetric care are crucial for teenage women residing in rural areas. Additionally, the majority of fistulas were simple fistulas and the most common type of OF was VVF. Surgical outcomes in the present study showed a high failure rate. Thus, collaboration with experienced teams in the management of urogenital fistulas could help us to improve our surgical outcomes.

### What is known about this topic

- *Obstetric fistula affects mostly young rural women of reproductive age after prolonged obstructed labor;*
- *Vesico-vaginal fistula is the most common type of urogenital fistulas;*
- *Generally, obstetric fistula repair has favorable surgical outcome in the hands of skilled surgeons*.

### What this study adds

- *In our setting, OF affects women of reproductive age, residing in rural areas, housekeeper and most often primiparous with a lack of antenatal care;*
- *OF occurs after spontaneous vaginal birth and prolonged obstructed labor;*
- *Failure rate is very high in our study*.
